# Under contract and in good health: a multigroup cross-lagged panel model of time use and health-related quality of life in working-age men and women

**DOI:** 10.1186/s12955-022-02052-3

**Published:** 2022-11-14

**Authors:** Laura Altweck, Samuel Tomczyk, Silke Schmidt

**Affiliations:** Department of Health and Prevention, University of Greifswald, Robert-Blum-Str. 13, 17489 Greifswald, Germany

**Keywords:** Time use, Health-related quality of life, Gender, Cross-lagged panel model, Longitudinal

## Abstract

**Background:**

Self-reported time-use in relation to health-related quality of life (HRQoL) has been widely studied, yet less is known about the directionality of the association and how it compares across genders when controlling for sociodemographic confounders.

**Methods:**

This study focused on the working population of the most recent waves (2013–2018) of the Core-Study of the German Socio-Economic Panel (*N* = 30,518, 46.70% female, *M* = 39.24 years). It examined the relationship between three time-use categories (contracted, committed, & leisure time) and HRQoL (self-rated health & life satisfaction) in men and women via multigroup fixed effects cross-lagged panel models. The models controlled for sociodemographic background (age, household income, number of children living in household, employment status, education, & marital status), which was associated with time-use and psychosocial health in previous research.

**Results:**

Contracted time showed consistent positive relationships with HRQoL across genders while associations with the other types of time use differed significantly between men and women and across indicators of HRQoL.

**Conclusions:**

The way we spend our time directly predicts our health perceptions, but in the same vein our health also predicts how we can spend our time. Contracted time in particular was associated with positive HRQoL, across genders, and beyond sociodemographic predictors, highlighting the important role of employment in health, for men and women alike. The impact of commitments beyond contracted time-use—like household chores and childcare—however, continues to affect mainly women, which ultimately reflects in poorer health outcomes.

**Supplementary Information:**

The online version contains supplementary material available at 10.1186/s12955-022-02052-3.

## Background

The association between time use and health is well-known [1, 2, 3]. For instance, spending more time in paid work and devoting more time to family and childcare can be associated with good health, but excessive time spent on housework can also lead to negative health outcomes [4]. The COVID-19 pandemic has also demonstrated a change in leisure activities with a decline in physical activity, and an increase in sedentary activities (e.g., screen-based), and in turn poorer mental health [5, 6, 7, 8]. Therefore, it is important to find and promote the right balance between demanding and relaxing activities that supports positive mental and physical functioning. To harmonize time-use research and support these efforts, scientists have developed categories of time use, to capture different types of time use and examine their association with health. In his seminal work, As [9] discerns *necessary* (e.g., hygiene), *contracted* (e.g., paid work), *committed* (e.g., housework), and *free time* (e.g., arts), which can be connected to subsequent health trajectories. This categorization has been utilized in multinational time-use surveys [10, 11], and connected to health. For instance, lower quality of life in unemployed persons with lower contracted time [12] or higher quality of life in persons with more artistic activities in their free time [13].

The present study examines time-use categories in relation to health-related quality of life (HRQoL), that is “perceived wellbeing in physical, mental, and social domains of health”([14], p. 195). The term HRQoL is used as the overall concept, while in the present analyses life satisfaction as well as self-rated health are examined. Sociodemographic and economic background—especially gender—has been shown to be an important predictor of HRQoL [15, 16].

In recent years, a gender perspective has enriched this research by asking questions about gender roles and ideologies, meaning individual (gender roles) and collective (gender ideologies) beliefs about the division of responsibilities and tasks between genders, for instance regarding work and family [17, 18, 19]. From a theoretical perspective, feminist theories, for instance, question the validity of traditional conceptualizations of work-family-balance with distinct roles of providers and carers, and propose more nuanced, gendered perspectives instead (e.g., reflecting on the symbolic meaning of unpaid housework as an act of love, and an equal contribution to paid work, [20, 21]). Individual perceptions of daily activities (e.g., childcare, housework) are also influenced by intra- and interindividual standards and expectations, which determine time and effort spent on a task, and subsequently positively or negatively evaluating said time use. In previous studies, women reported higher standards regarding housework and were often disappointed by their male partners, which led to marital distress and the women completing these tasks [21]. Performing said tasks can also be described as doing-gender [22], with women fulfilling expectations and social norms. Other approaches like the marital-power framework associates an imbalance of resources favouring men (e.g., via institutional preference in the workplace) with a power imbalance in marriages that leads to women performing more undesirable activities like housework (e.g., [17]). Further analysis of gender perceptions reveals differing preferences and interpretations of roles (e.g., within the workplace, social settings, and families that directly or indirectly affect time use) and psychosocial health, for example, valuing social integration over career success corresponds to social expectations towards women but can lead to role disengagement at work and thus foster work-family conflict [23]. Social perspectives and institutional, and thereby, societal structures are intertwined with individual perceptions and actions regarding time use. In a study on gender and career success, Frear et al. [24] found support for both the unequal attributes model (i.e., differing attributes like job status, family-related values, and personal resources mediate gender differences in career success) as well as the unequal effects model (i.e., gender moderates the impact of attributes on career success). These findings imply that both, institutional contexts as well as individual expectations and resources affect career success and consequently determine time spent on career development (e.g., contracted time). Overall, current research directions range from cross-cultural comparisons of ideologies (e.g., egalitarian, traditional) and their association with work-family policies [25] to studies on individual perceptions [24]. Regarding time-use research, the association of gender, time-use patterns, and health is complicated: For instance, higher levels of multitasking work, housework, and childcare in women compared to men are related to lower happiness, life satisfaction, and quality of life [19, 26, 27, 28]. In essence, unpaid care work has been identified as a core aspect of gender inequality across countries ([4, 17, 18, 29, 30, 31]), and it is associated with negative emotions and psychological distress, particularly in women [32, 33]. In line with feminist theory, this could symbolize an imbalance of resources with women receiving insufficient esteem or not being sufficiently valued for their contributions [20, 21]. Women also have less time for personal care and leisure activities [34] that limits the self-regulatory potential of time use to achieve life-domain balance [23]. Moreover, men are less likely to reduce working hours to increase time spent on childcare to alleviate their partner’s stress levels [30], which might lead to further conflict and stress. It is important to note that these effects vary based on the operationalization of time use. In a large meta-analysis, Shockley et al. [35] did not find strong empirical support for gender differences, yet they focused on work-family conflict (as one specific subtype of gendered time use) and did not examine country-level effects, like national policies.

To reiterate, the gendered association between time use and health is strongly affected by sociodemographic characteristics and societal context. For instance, becoming unemployed and unemployment status affect men’s wellbeing more negatively than women’s [36, 37, 38], which is linked to their contracted time. Higher contracted time is also associated with less necessary time (e.g., sleep, self-care) but better self-rated health across countries [2]. Gender policies on a national level—e.g., regarding childcare—also influence gendered time use: in Sweden and other Scandinavian countries, time spent on work and childcare is more similar in men and women than in Italy [39, 40]. Despite a large body of research in this area, few studies have prospectively investigated the interplay of gender, sociodemographic factors (e.g., employment status), time use, and psychosocial health in population-based samples. To date, many studies are either cross-sectional, assess only one aspect of time use (e.g., contracted time) or focus on a single socio-economic factor (e.g., income, employment status) in relation to gender. Moreover, bidirectional effects (i.e., of health status on time-use and vice versa) also need further attention.

To add to the literature, this study presents a longitudinal analysis of panel data to answer the following research questions: How does the relationship between types of time use and HRQoL compare across genders? Which bidirectional effects emerge over time? And, which of these associations are significant above and beyond controlling for sociodemographic variables?

## Material and methods

### Data set and ethical considerations

Data from the Core-Study of the German Socio-Economic Panel (GSOEP) was used. This is an annual representative longitudinal study of private households from 1984 until present [41, 42]. The GSOEP is a fixed panel—i.e., the same persons are surveyed annually—but refresher samples of new persons are also regularly introduced to compensate drop-out. We used the waves which measured time use (i.e., 2013–2018, *N* = 89,161), and restricted the sample to participants of working age (i.e., 18–65 years; *n* = 30,518).

This study is a secondary data analysis; therefore, no additional ethical approval was needed. See declarations for details.

### Variables and measures

#### Time-use categories

Time use was measured with the question “What is a typical weekday like for you? How many hours per normal workday do you spend on the following activities?”: (1) *work*, apprenticeship (including travel time to and from work), (2) *education* or further training (also school/university), (3) *errands* (shopping, trips to government agencies, etc.), (4) *housework* (washing/cooking/cleaning), (5) *childcare*, (6) *care* and support of persons in need of care, (7) *repairs* on and around the home or car, and *garden* or lawn work, (8) *physical activity* (sport, fitness, gymnastics), (9) *hobbies* and other *leisure*-time activities, and (10) *sleep*. Participants were allowed to answer freely, including giving fractions of hours. Activities were categorized in accordance with As’ [9] classification system, who differentiated between contracted, committed, free, and necessary time. *Contracted time* included the items *work*, and *education*. *Committed time* included the items *errands*, *housework*, *childcare*, *care*, and *repairs*. *Free time* included the items *physical activity*, and *hobbies*. We only focused on these three time-use categories because the GSOEP only measures one aspect of the category *necessary time* (namely sleep), other aspects like eating or self-care were not assessed. Items were averaged for each category.

#### Health-related quality of life

*Self-rated health (SRH)* was measured using the item “How would you describe your current health?”. The 5-point scale—*1* (very good) to *5* (bad)—was inverted so that higher values reflected better self-rated health. *Life satisfaction (LS)* was measured using the items „How satisfied are you with your life overall?” [43]. An 11-point scale of *0* (completely dissatisfied) to *10* (completely satisfied) was used, where higher values reflected greater life satisfaction.

#### Socio-demographic variables

*Gender* was used as a grouping variable in the cross-lagged panel models and was dummy coded (1 = female, 0 = male). The other sociodemographic variables were treated as confounders, as time use and HRQoL vary across a number of sociodemographic variables [36, 37]. As our focus lies on gender differences in time use and HRQoL, which can be explained, for instance, by differences in gender roles, balancing work and family life, and ultimately male versus female norms (e.g., [23]) and following previous analyses [4] we included the following confounder variables: *age* (years), *household income* (Euros ÷ 1000 per month), *number of children living in household*, *employment status* (dummy coded; no employment [reference category], part-time employment, full-time employment), *education* (dummy coded ISCED 2011: low = 0–2 [reference category], middle = 3–4, high = 5–8), and *marital status* (1 = married, 0 = not married). Data of the sociodemographic variables came from the 2013 wave.

### Statistical analysis

#### Descriptive statistics

We examined missing data patterns and distributions of the analysis data set by calculating mean values, standard deviations for continuous variables, and relative frequencies for categorical variables. Data was compared between genders, and subsequently via t-tests, ANOVA, and chi square tests.

#### Cross-lagged panel models

**Fig. 1:**
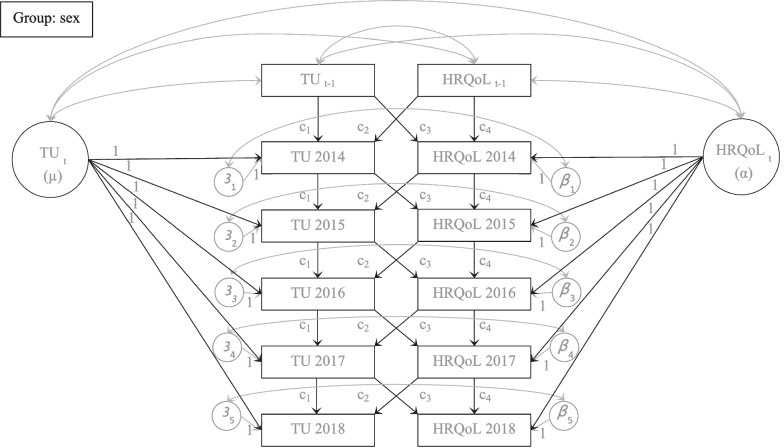
Multigroup cross-lagged panel model of time-use category and HRQoL. *Note.* TU: time use category. HRQoL: health-related quality of life. Correlations are depicted in grey. Autoregressive and cross-lagged paths (c_1-4_) were constrained to be equal across time

To examine the longitudinal cross lagged effects between time-use categories and HRQoL across gender we employed multigroup (MG) fixed effects cross-lagged panel models (CLPMs; [44], see Fig. 1). This approach treats the baseline values (*t*_*-1*_ = 2013) of *x* (time-use categories) and *y* (HRQoL) as predetermined. The autoregressive and cross-lagged paths were constrained to be equal across time points [44], so that fixed effects for the following waves of *x* and *y* were introduced as latent variables (‘2014–2018 *x*’→*µ* & ‘2014–2018 *y*’→*α*) into the models [44]. To test model difference across gender, unconstrained and constrained models were run, namely where the paths of interest were allowed to vary freely or constrained to be equal across gender. Following the approach of Allison et al. [45] the baseline sociodemographic variables were regressed onto the baseline and latent variables of time-use categories and HRQoL.

The robust maximum likelihood (ML) estimator and—to handle missing data—full information maximum likelihood (FIML) method were used. We modelled six MG CLPMs for each type of time use (contracted, committed, & free time), and HRQoL (LS & SRH) separately (see Additional file 1: sample code). Due to multiple testing, the Bonferroni correction was applied (*p* ≤ 0.0008 [0.05 ÷ 6]).

The Comparative Fit Index (CFI) > 0.95, Root Mean Squared Error of Approximation (RMSEA) < 0.06, and Standardized Root Mean Square Residual (SRMR) < 0.08 were used to evaluate model fit [46]. Models were compared using the chi square difference test and were ranked ordered using the Bayesian Information Criterion (BIC), and the Akaike Information Criterion (AIC), where lower values indicate better models [47].

**Fig. 2:**
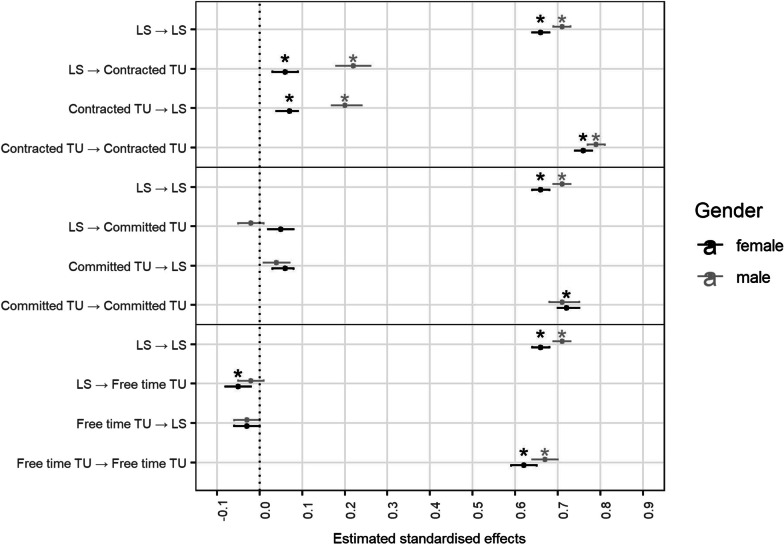
Multigroup cross-lagged panel model results for life satisfaction (standardised values). *Note.* *p ≤ .0008. LS: life satisfaction, TU: time use

**Fig. 3:**
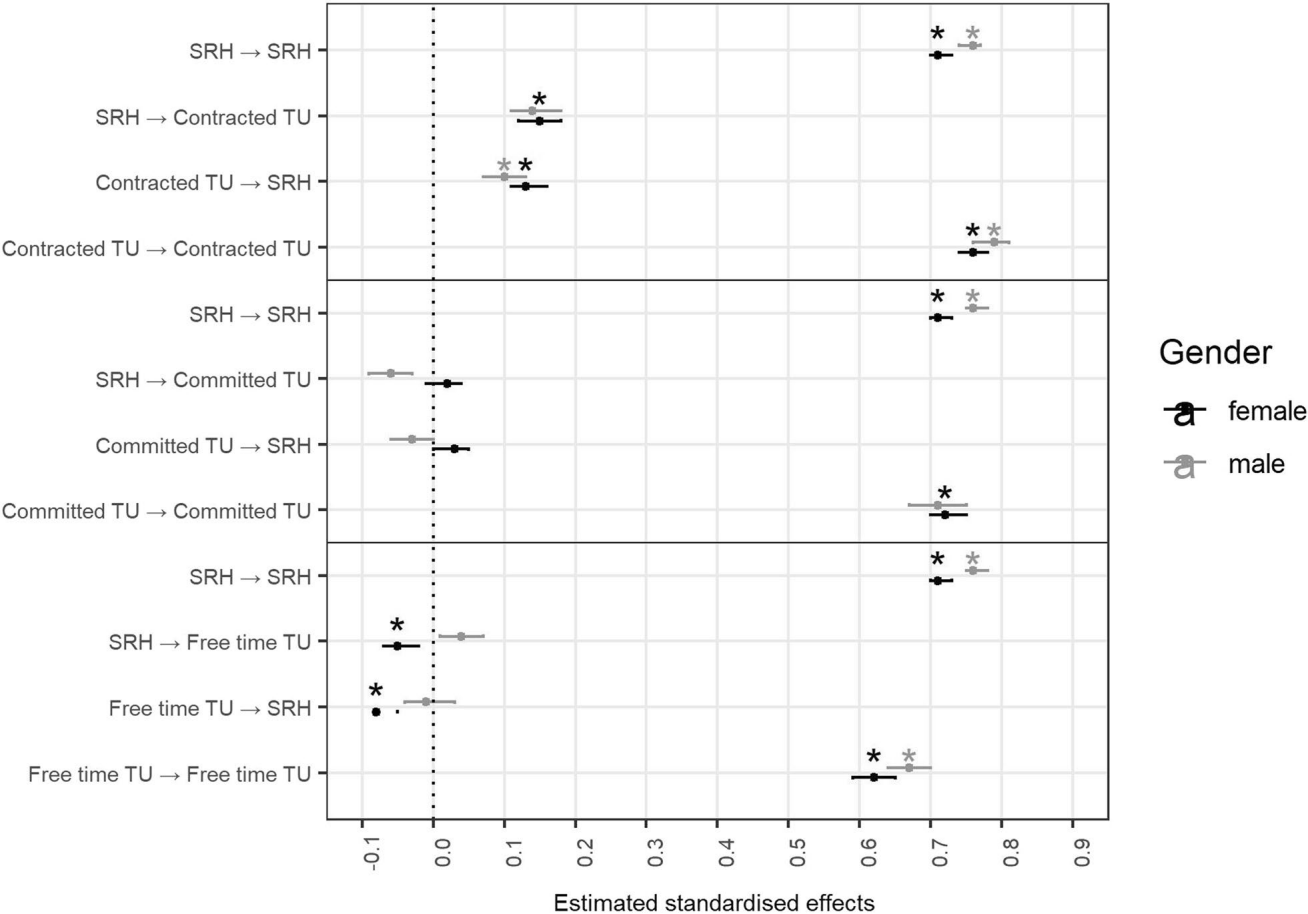
Multigroup cross-lagged panel model results for self-rated health (standardised values). *Note.* *p ≤ .0008. SRH: self-rated health, TU: time use

The statistical program *R* version 3.6.2 [48] was used for all analyses, along with the packages *car* (v3.0–10, [49]) and *tidyverse* (v1.3.0, [50]) to recode variables, *lavaan* (v0.6–7, [51]) to run the MG CLMPs, *DescTools* (v0.99.43, [52]) to summarise the output, and *ggplot2* (v3.3.3, [53]) to create Figs. 2 and 3. This study was not preregistered.

## Results

### Descriptive statistics

The sample comprised 30,518 participants (46.70% female; see Table 1). In 2013, the mean age was 39.24 years (*SD* = 11.60), about half the sample had completed middle education (55.94%), were in full-time employment (52.71%), and married (56.55%), had an average household income of €3,125.12 (*SD* = 2,037.37), and one child in the household (*M* = 1.07, *SD* = 1.19). Women reported a significantly lower household income, more children in the household, were more likely to hold middle education but less likely to have completed higher education, to be in non-traditional employment, and to be married than men.

**Table 1:** Descriptive statistics of sample: total, and by gender (*n* = 30,518)

	Total		Female		Male		Difference
	*M*/*n*	*SD*/%	*M*/*n*	*SD*/%	*M*/*n*	*SD*/%	test
Age (years)	39.24	11.60	39.18	11.41	39.30	11.82	
HHI (€ ÷ 1000)	3.13	2.04	3.05	2.03	3.22	2.04	***
Children	1.07	1.19	1.09	1.17	1.05	1.21	*
Education							
Low ○	3956	16.96	2198	17.02	1758	16.89	
Middle	13,046	55.94	7489	57.99	5557	53.39	***
High	6321	27.10	3227	24.99	3094	29.72	***
Employment status							
Non-traditional○	5769	27.20	3921	34.43	1848	18.81	
Part-time	4261	20.09	3818	33.52	443	4.51	***
Full-time	11,181	52.71	3650	32.05	7531	76.67	***
Marital status							
Not ○	10,330	43.45	5945	45.25	4385	41.23	
Married	13,443	56.55	7193	54.75	6250	58.77	***
	30,518	100.00	14,252	46.70	16,266	53.30	

*Note*. Test significance: *** *p* < .001, ** *p* < .01, * *p* < .05. HHI: household income. Children: number of children living in household. Non-traditional work includes maternity leave, retirement, or being a student. ○ reference group. Sociodemographic data from wave 2013

See Additional file 2 for descriptive statistics and correlations of time-use categories and HRQoL. From 2013–2018 women reported significantly more committed time use (*M*_w_ = 1.75–2.08, *M*_m_ = 0.79–0.92, *p* < 0.001), while men reported more contracted time use (*M*_m_ = 3.35–4.28, *M*_w_ = 2.50–2.85, *p* < 0.001), and free time (*M*_m_ = 0.94–1.10, *M*_w_ = 0.91–1.04, *p* < 0.05–0.001). Women reported slightly higher LS across all waves (*M*_w_ = 7.26–7.42, *M*_m_ = 7.24–7.41, *p* < 0.001) but lower SRH in recent years (2016–2018: *M*_w_ = 3.46–3.47, *M*_m_ = 3.63–3.64, *p* < 0.01-0.001).

### Multigroup cross-lagged panel models

The model fit indices indicated excellent fit for all models. First, we examined the MG CLPMs without control variables (see Table 2, Figs. 2 and 3, and Additional file 3). Significant positive bidirectional cross-lagged effects between contracted time use and LS were seen in both genders, where the effects were stronger in the male sample. Significant positive bidirectional cross-lagged effects between contracted time use and SRH were also seen in both genders, here slightly larger effects were seen in the female sample. Only in the female sample more committed time use significantly predicted greater LS, while only in the male sample greater SRH predicted lower committed time use. In contrast, only in the female sample greater LS predicted lower free time, and significant negative bidirectional cross-lagged effects between free time and SRH were seen.

**Table 2:** Model fit indices of the time use and health-related quality of life cross-lagged panel models

	Model fit	(Δ) Chi Sq	(Δ) df	*p*	CFI	SRMR	RMSEA	AIC	BIC
*Contracted time use ↔ Life satisfaction*									
*n *_*female*_* | n *_*male*_	Unconstrained	1818.56	130	0.0000	0.99	0.03	0.03	931,679.78	932,107.69
19,933 | 18,560	Constrained	67.03	4	0.0000	0.98	0.03	0.03	931,781.64	932,175.31
*Committed time use ↔ Life satisfaction*									
*n *_*female*_* | n *_*male*_	Unconstrained	2882.43	130	0.000	0.97	0.04	0.03	836,044.24	836,472.13
19,924 | 18,550	Constrained	1268.65	4	0.000	0.96	0.07	0.04	837,521.88	837,915.54
*Leisure time ↔ Life satisfaction*									
*n *_*female*_* | n *_*male*_	Unconstrained	2991.07	130	0.000	0.96	0.04	0.03	787,085.83	787,513.75
19,934 | 18,560	Constrained	84.99	4	0.000	0.96	0.05	0.03	787,220.15	787,613.83
*Contracted time use ↔ Self-rated health*									
*n *_*female*_* | n *_*male*_	Unconstrained	1696.22	130	0.000	0.99	0.03	0.03	765,894.46	766,322.38
19,935 | 18,561	Constrained	17.63	4	0.001	0.99	0.03	0.02	765,912.61	766,306.29
*Committed time use ↔ Self-rated health*									
*n *_*female*_* | n *_*male*_	Unconstrained	2556.11	130	0.000	0.98	0.04	0.03	669,752.47	670,180.38
19,933 | 18,560	Constrained	1417.91	4	0.000	0.96	0.07	0.04	671,219.21	671,612.89
*Leisure time ↔ Self-rated health*									
*n *_*female*_* | n *_*male*_	Unconstrained	2739.26	130	0.000	0.97	0.04	0.03	620,752.79	621,180.70
19,934 | 18,562	Constrained	105.22	4	0.000	0.97	0.05	0.03	620,899.97	621,293.65

*Note* Chi Sq: chi square. In constrained models the four paths were constrained to be equal across genders

*Note* Significant effects *p* ≤ .0008 are boldened

When control variables were entered into the MG CLPMs most of the cross-lagged paths between time-use category and HRQoL became non-significant (*p* ≥ 0.0008; see Table 3 and Additional file 4). However, the significant positive bidirectional, cross-lagged effects between contracted time use and SRH remained across genders, where the effects were now stronger in the male sample. Also, higher committed time use now predicted lower SRH in the female sample.

**Table 3:** Multigroup cross-lagged panel model results, controlling for socio-demographic variables (standardised values)

				Female				Male			
				ß	LB	HB	*p*	ß	LB	HB	*p*
	LS	→	LS	**0.61**	**0.59**	**0.63**	**0.0000**	**0.65**	**0.63**	**0.67**	**0.0000**
	LS	→	Contracted TU	− 0.01	− 0.04	0.02	0.3574	0.03	− 0.01	0.06	0.1561
	Contracted TU	→	LS	0.02	− 0.01	0.05	0.2683	0.05	0.01	0.09	0.0133
	Contracted TU	→	Contracted TU	**0.49**	**0.45**	**0.52**	**0.0000**	**0.49**	**0.46**	**0.53**	**0.0000**
Model / fit	Chi Sq	df	*p*	cfi	SRMR	rmsea	AIC	BIC	Δ Chi Sq	Δ df	*p*
Unconstrained	1509.95	258	0.0000	0.99	0.02	0.02	562,325.84	563,225.85			
Constrained	1521.20	262	0.0000	0.99	0.02	0.02	562,329.09	563,197.52	7.29	4	0.1215
	LS	→	LS	**0.61**	**0.59**	**0.63**	**0.0000**	**0.65**	**0.63**	**0.67**	**0.0000**
	LS	→	Committed TU	− 0.01	− 0.04	0.02	0.3574	0.03	− 0.01	0.06	0.1561
	Committed TU	→	LS	0.02	− 0.01	0.05	0.2683	0.05	0.01	0.09	0.0133
	Committed TU	→	Committed TU	**0.49**	**0.45**	**0.52**	**0.0000**	**0.49**	**0.46**	**0.53**	**0.0000**
Model / fit	Chi Sq	df	*p*	cfi	SRMR	rmsea	AIC	BIC	Δ Chi Sq	Δ df	*p*
Unconstrained	2564.75	258	0.0000	0.97	0.03	0.03	547,423.19	548,323.20			
Unconstrained	3169.49	262	0.0000	0.97	0.04	0.03	548,019.92	548,888.36	371.59	4	0.0000
	LS	→	LS	**0.61**	**0.59**	**0.63**	**0.0000**	**0.65**	**0.63**	**0.67**	**0.0000**
	LS	→	Free time TU	0.00	− 0.03	0.03	0.8895	0.02	− 0.01	0.05	0.2406
	Free time TU	→	LS	0.02	− 0.01	0.05	0.1941	0.04	0.00	0.08	0.0396
	Free time TU	→	Free time TU	**0.56**	**0.53**	**0.60**	**0.0000**	**0.58**	**0.54**	**0.62**	**0.0000**
Model / fit	Chi Sq	df	*p*	cfi	SRMR	rmsea	AIC	BIC	Δ Chi Sq	Δ df	*p*
Unconstrained	1775.20	258	0.0000	0.98	0.02	0.02	489,954.16	490,854.18			
Constrained	1795.89	262	0.0000	0.98	0.02	0.02	489,966.85	490,835.29	12.22	4	0.0158
	SRH	→	SRH	**0.66**	**0.64**	**0.67**	**0.0000**	**0.69**	**0.67**	**0.71**	**0.0000**
	SRH	→	Contracted TU	**0.06**	**0.03**	**0.09**	**0.0001**	**0.10**	**0.06**	**0.13**	**0.0000**
	Contracted TU	→	SRH	**0.09**	**0.06**	**0.13**	**0.0000**	**0.13**	**0.09**	**0.17**	**0.0000**
	Contracted TU	→	Contracted TU	**0.49**	**0.45**	**0.52**	**0.0000**	**0.50**	**0.46**	**0.53**	**0.0000**
Model / fit	Chi Sq	df	*p*	cfi	SRMR	rmsea	AIC	BIC	Δ Chi Sq	Δ df	*p*
Unconstrained	1386.26	258	0.0000	0.99	0.02	0.02	452,856.77	453,756.79			
Constrained	1391.32	262	0.0000	0.99	0.02	0.02	452,853.84	453,722.27	3.58	4	0.4662
	SRH	→	SRH	**0.66**	**0.64**	**0.68**	**0.0000**	**0.69**	**0.67**	**0.71**	**0.0000**
	SRH	→	Committed TU	− 0.02	− 0.06	0.01	0.1148	− 0.01	− 0.04	0.02	0.4647
	Committed TU	→	SRH	**− 0.06**	**− 0.09**	**− 0.03**	**0.0002**	− 0.05	− 0.08	− 0.01	0.0049
	Committed TU	→	Committed TU	**0.58**	**0.55**	**0.62**	**0.0000**	**0.63**	**0.58**	**0.68**	**0.0000**
Model / fit	Chi Sq	df	*p*	cfi	SRMR	rmsea	AIC	BIC	Δ Chi Sq	Δ df	*p*
Unconstrained	2445.10	258	0.0000	0.98	0.03	0.03	438,179.19	439,079.20			
Constrained	3043.34	262	0.0000	0.97	0.04	0.03	438,769.44	439,637.87	391.67	4	0.0000
	SRH	→	SRH	**0.66**	**0.64**	**0.68**	**0.0000**	**0.69**	**0.67**	**0.71**	**0.0000**
	SRH	→	Free time TU	− 0.01	− 0.04	0.02	0.5707	0.02	− 0.01	0.06	0.1581
	Free time TU	→	SRH	− 0.01	− 0.04	0.02	0.6629	0.00	− 0.03	0.04	0.8113
	Free time TU	→	Free time TU	**0.56**	**0.53**	**0.60**	**0.0000**	**0.58**	**0.54**	**0.62**	**0.0000**
Model / fit	Chi Sq	df	*p*	cfi	SRMR	rmsea	AIC	BIC	Δ Chi Sq	Δ df	*p*
Unconstrained	1687.63	258	0.0000	0.98	0.02	0.02	380,737.69	381,637.71			
Constrained	1703.04	262	0.0000	0.98	0.02	0.02	380,745.10	381,613.54	10.18	4	0.0375

## Discussion

By means of MG CLPMs, this study examined the bidirectional effects of contracted, committed, as well as leisure time and HRQoL in men and women. We found that contracted time showed consistent positive relationships with HRQoL across genders while associations with the other types of time use differed significantly in men and women and across models.

Similar to previous work (e.g., [2]), we also found that more contracted time related to better life satisfaction and self-rated health. This held across gender, giving credence to research finding greater similarities rather than differences in the importance of the link between contracted time use and HRQoL in men and women [35]. With a trend towards increasingly greater endorsement of egalitarian gender roles in past decades [54], the importance of the life domains education and employment for women’s health appears to have aligned with men’s. The increased importance of contracted time for men [36, 37], however, also seems to be visible in our analysis, as the associations in covariate-adjusted CLPMs were stronger for men than for women.

Noticeably, while men—by far—still spent most of their day with contracted time use, women reported essentially equal hours between contracted and committed time use. To no surprise then, committed and leisure time showed stronger effects with HRQoL in women than men. This gives credence to the perspective of gender inequality in time allocation and health [19, 27, 30], which highlights that the burden of balancing paid work, housework, and childcare—along with its detrimental costs to health—still mainly lies on women [21]. This goes beyond previous research on work-family conflict because the analysis differentiates between leisure activities, committed (family) time, and contracted time, and their differential impact on health. The findings further support the unequal effects model [24], since associations between time use and health differ between genders, when controlling for potentially divergent attributes. For example, while participating in hobbies has been found to provide health benefits and relief from the stressful day to day [13, 55], we found that greater leisure time was related to lower HRQoL in women. Thus, this potential benefit appears to mainly apply to men. Instead, leisure time may be a—lacking—luxury for women [34], which serves as a reminder of allocating time to more ‘necessary’ domains (e.g., work, childcare, errands). On the other hand, men who reported greater self-rated health allocated less time to committed time, which can be interpreted as their priorities lying elsewhere (e.g., work) and devoting less time to supporting their spouses—which corroborates previous findings [30].

Apart from gender, the way we spend our time is also strongly influenced by other sociodemographic variables, such as education or income [4]. Previous studies examining time use often did not consider the confounding effects of sociodemographic background (e.g., [10, 11]), which may in part explain inconsistent findings on gender effects. Furthermore, economic factors have been shown to influence health and time use [56], for example, a mother with more financial savings is likely at greater liberty to not have to work full-time and spend more time with her children. In this study, we controlled for sociodemographic variables in the MG CLPMs. Indeed, we found that most associations between time use, and health were explained by sociodemographic differences (e.g., employment, marital, or education status). However, the bidirectional effects between higher contracted time use and greater self-rated health remained above and beyond, indicating a particularly unique effect. Further, while more committed time use in women predicted greater life satisfaction in models without covariates, only when sociodemographic factors were accounted for, the negative health effects were revealed. It seems that sociodemographic effects could mask gender effects, which is why future research should take a closer look at interactions with sociodemographic (as well as economic) variables when examining time-use patterns and health trajectories.

## Strengths, limitations, and future directions

Our study used a large a population sample to examine bidirectional effects of time use and HRQoL, where previous studies did not consider the bidirectional relationship [10, 11]. Second, our approach allowed us to determine that time use was related to HRQoL above and beyond sociodemographic background, lending credence to the study of time use.

Naturally this study is not without its limitations. We used a representative sample, which captured self-reports on the subjective experience of time use and HRQoL. Thus, our results might be affected by recall bias regarding time-use measures, and method bias due to a single source. To address these limitations, future studies may want to use objective measures to capture time use (e.g., smart watch), and HRQoL (e.g., absence from work due to sickness, health diagnoses, or health insurance data).

Also, the items measuring free time are unable to distinguish between activities, which is noteworthy as the literature shows differences with health outcomes (physically active vs. inactive, alone or in company; e.g., [8]).

The present statistical approach focused on the importance of interpersonal (gender) differences—neglecting intrapersonal differences across time—however, this aspect is especially relevant for research questions where larger period effects are expected in time use and HRQoL (e.g., before, during and after the COVID-19 pandemic). Further, by examining LS and SRH in separate models we were able to distinguish between time-use effects on different aspects of HRQoL, but the approach also neglected the correlations between these constructs.

Our findings are also limited to the German population, which is known for its close entanglement of identity and work [57]. It will be interesting to see whether the findings translate to other countries and cultural settings, where gender policies and views towards gender roles differ (e.g., [25]). We also acknowledge that the scope of our analysis regarding gender is limited and does not account for multidimensional effects, for instance, on individual (gender roles) and collective (gender ideologies and policies) levels. Future research should thus utilize multidimensional frameworks to examine gender-based effects on time use, and health.

## Conclusions

The way we spend our time directly predicts our health perceptions, but in the same vein our health also predicts how we can spend our time. This analysis shows that contracted time in particular is associated with positive HRQoL, across genders, and beyond sociodemographic background. This speaks for the important role of employment in health, for men and women alike. The need to balance work with household chores, childcare, and the like, however, seems to mainly affect women, at least in Germany, which ultimately reflects in poorer health outcomes. Adding to the research on work-family conflict, this study further differentiates non-work activities, considers sociodemographic confounders of bidirectional, longitudinal associations, and thus presents new implications for the long-standing debate on gender, time use, and health.

## Supplementary Information

**Additional file 1.** R sample code.

**Additional file 2.** Descriptive statistics and correlations of time-use categories and HRQoL.

**Additional file 3.** Full results of the multigroup cross-lagged panel model without controls.

**Additional file 4.** Full results of the multigroup cross-lagged panel model with controls.

## Data Availability

The data can be accessed following an application to the German Institute for Socioeconomic research: https://www.diw.de/en/diw_01.c.618953.en/the_institute.html
